# The role of cannabinoids in pain modulation in companion animals

**DOI:** 10.3389/fvets.2022.1050884

**Published:** 2023-01-04

**Authors:** Agatha Miranda-Cortés, Daniel Mota-Rojas, Nadia Crosignani-Outeda, Alejandro Casas-Alvarado, Julio Martínez-Burnes, Adriana Olmos-Hernández, Patricia Mora-Medina, Antonio Verduzco-Mendoza, Ismael Hernández-Ávalos

**Affiliations:** ^1^Department of Biological Science, Clinical Pharmacology and Veterinary Anesthesia, Universidad Nacional Autónoma de México (UNAM), FESC, Mexico City, Mexico; ^2^Neurophysiology of Pain, Behavior and Assessment of Welfare in Domestic Animals, DPAA, Universidad Autónoma Metropolitana, (UAM), Mexico City, Mexico; ^3^Department of Clinics and Veterinary Hospital, School of Veterinary, University of Republic, Montevideo, Uruguay; ^4^Animal Health Group, Facultad de Medicina Veterinaria y Zootecnia, Universidad Autónoma de Tamaulipas, Ciudad Victoria, Tamaulipas, Mexico; ^5^Department Bioterio and Experimental Surgery, Instituto Nacional de Rehabilitación-Luis Guillermo Ibarra Ibarra (INR-LGII), Calzada México Xochimilco, Mexico City, Mexico; ^6^Livestock Science Department, Universidad Nacional Autónoma de México (UNAM), FESC, Mexico City, Mexico

**Keywords:** analgesia, animal welfare, cannabinoid receptors, endocannabinoid system, marijuana

## Abstract

The use of cannabinoids in both veterinary and human medicine is controversial for legal and ethical reasons. Nonetheless, the availability and therapeutic use of naturally occurring or synthetic phytocannabinoids, such as Δ^9^-tetrahydrocannabidiol and cannabidiol, have been the focus of attention in studies regarding their medical uses. This review aims to examine the role of cannabinoids in pain modulation by analyzing scientific findings regarding the signaling pathways of the endocannabinoid system and discussing the analgesic effects of synthetic cannabinoids compared to cannabinoid extracts and the extent and involvement of their receptors. In animals, studies have shown the analgesic properties of these substances and the role of the cannabinoid binding −1 (CB1) and cannabinoid binding −2 (CB2) receptors in the endocannabinoid system to modulate acute, chronic and neuropathic pain. This system consists of three main components: endogenous ligands (anandamide and 2-arachidonoylglycerol), G protein-coupled receptors and enzymes that degrade and recycle the ligands. Evidence suggests that their interaction with CB1 receptors inhibits signaling in pain pathways and causes psychoactive effects. On the other hand, CB2 receptors are associated with anti-inflammatory and analgesic reactions and effects on the immune system. Cannabis extracts and their synthetic derivatives are an effective therapeutic tool that contributes to compassionate pain care and participates in its multimodal management. However, the endocannabinoid system interacts with different endogenous ligands and neurotransmitters, thus offering other therapeutic possibilities in dogs and cats, such is the case of those patients who suffer from seizures or epilepsy, contact and atopic dermatitis, degenerative myelopathies, asthma, diabetes and glaucoma, among other inflammatory diseases. Moreover, these compounds have been shown to possess antineoplastic, appetite-stimulating, and antiemetic properties. Ultimately, the study of the endocannabinoid system, its ligands, receptors, mechanism of action, and signaling, has contributed to the development of research that shows that hemp-derived and their synthetic derivatives are an effective therapeutic alternative in the multimodal management of pain in dogs and cats due to their ability to prevent peripheral and central sensitization.

## Introduction

The use of cannabinoids in human and veterinary medicine has been controversial for ethical and legal reasons. Despite this, hemp-derived compounds are gaining medical approval for their benefits. However, drug use is complicated by the application of laws and professional regulations of each country (1), so further research is needed to document and support their clinical use (2). Cannabinoids are a group of compounds obtained from hemp (*Cannabis sativa L*.) that have been used for different therapeutic purposes in dogs, cats, and ferrets (2, 3): antispastics, antiemetics, anticonvulsants, and appetite stimulants, or for their neuroprotective, analgesic and anti-inflammatory properties. Other documented uses in rodent models are as a treatment for cancer, asthma, diabetes, and retinitis pigmentosa (3). They also could be effective in managing pain related to osteoarthritis (2–4), which has prompted owners of companion animals to use cannabinoids as a natural alternative with potential benefits (5).

Several controlled clinical trials were initiated over 20 years ago to treat various pathologies using cannabinoids. These substances have been used to treat health problems such as cephalgia, fever, bacterial infections, diarrhea, rheumatic pain, or malaria (4, 6). These resulted in the approval of cannabis-based products such as dronabinol, nabilone, and an extract of delta-9-tetrahydrocannabinol (Δ^9^-THC) and cannabidiol (CBD) (7). The total synthesis of (-) and (+) optical isomers was first published in 1965 (8). Later in 1988, after the discovery of the first endogenous cannabinoids, several studies were carried out that described the participation of the endocannabinoid system to control nausea and pain related to cancer or to reduce these signs produced by antineoplastic treatments (9, 10).

This review aims to analyze and describe the pharmacokinetic and pharmacodynamic characteristics of cannabinoids and the spatial distribution of their receptors, focusing on their role in the modulation of pain in companion animals. It will discuss and compare the analgesic effect of synthetic cannabinoids and extracts and analyze scientific findings regarding the endocannabinoid system's signaling pathways and its receptors' range and participation.

## Chemical structure

Cannabinoids have a carboxylic chemical structure with 21 carbons consisting of three rings: cyclohexane, tetrahydropyran, and benzene (10, 11). To study cannabinoids, researchers have classified them as chemical substances that interact with specific receptors, divided into three groups: herbal cannabinoids (phytocannabinoids), endogenous cannabinoids (endocannabinoids), which can be found in human or animal organisms, and synthetic cannabinoids (3, 10, 12). The word tetrahydrocannabinol (THC) is generally used to designate the (–)-trans-Δ^9^-tetrahydrocannabinol isomer (dronabinol, previously 1-, 3,4-trans-tetrahydrocannabinol), which can be linked to most pharmacological effects of cannabis, including its psychoactive properties. On the other hand, CBD is the most studied important non-psychotropic found in cannabis (13, 14).

To date, 489 chemical compounds have been identified, 70 belonging to the phytocannabinoids group and its subcategories. THC and CBD have been particularly relevant in pharmacological therapy. Δ^9^-THC has a tri-cyclic 21- carbon structure without nitrogen and two chiral centers in trans configuration; it is volatile viscous oil with high lipid solubility and low aqueous solubility, and a pKa of 10.6 (15). Δ^9^-THC has been shown to interact with type 1 and 2 receptors, with a particular affinity with type 2 receptors. In contrast, CBD has a lesser affinity to both receptors (16–18). The chemical name of CBD is 5'methyl-2'-(prop-1-en-2yl)-1',2',3',4'-tetrahydro-1.1'-biphenyl]-2,6-dioles retaining the trans- (1R,6R) (19).

On a physiological level, endocannabinoids act as endogenous ligands for the anandamide and 2-arachidonoylglycerol (2-AG) receptors, which regulate and modulate nociception, lipid metabolism, and gastrointestinal, cardiovascular, and motor functions (19, 20). Synthetic analogs have a similar structure to phytocannabinoids and are synthesized to mimic their effects (21). As their use increases, more studies have tried to manufacture different products for medical purposes, such as HU-210, considered a CB1 and CB2 agonist (3, 14, 22).

## Pharmacokinetic characteristics

**Figure 1:**
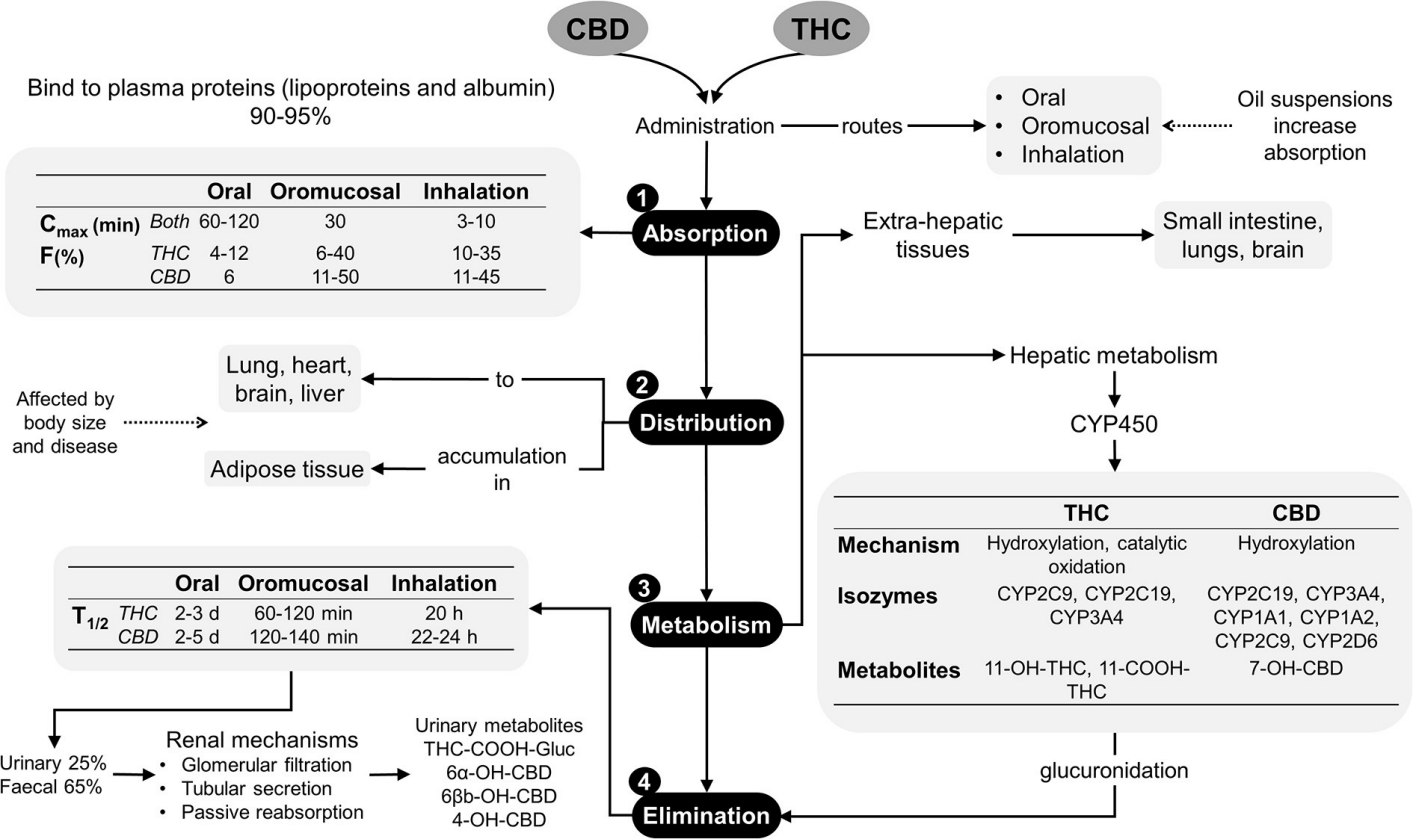
Pharmacokinetics of phytocannabinoids (10, 18, 29). CBD, cannabidiol; CYP450, cytochrome P450; d, days; F%, bioavailability; h, hours; min, minutes; T_1/2_, elimination half-life; THC, delta-9-tetrahydrocannabinol.

In dogs and cats, cannabinoid pharmacokinetic studies suggest administering different doses of THC, CBD, or products that contain both phytocannabinoids by oral, transmucosal, transdermal, intravenous and sublingual routes, where the majority of these products are made from hemp (12, 23, 24). The main phytocannabinoids reported are terpenes, flavonoids, CBD, cannabidiolic acid (CBDA) and THC, which should be considered as the main factor influencing the evaluation of a pharmacokinetic response since most products do not have a commercial standardization available for regulation and investigation. Despite this limitation, there is sufficient evidence of its pharmacokinetic characteristics (25). *Absorption*. Cannabinoids are easily absorbed due to their hydrophobic and liposoluble properties (11), although they are difficult to excrete without significant biotransformation. *Distribution*. Lipophilic drugs make a second plasma peak common due to slow gastric emptying, redistribution, and even different absorption windows in the gastrointestinal tract. When these substances are administered orally or intravenously, they bind to lipoproteins, albumin, and erythrocytes once in the bloodstream. Cannabinoids can bind to CB1 and CB2 receptors in the Central Nervous System (CNS) and the Peripheral Nervous System (PNS), and the effects can be seen 0.5 to 2 h after being administered (26, 27). In addition, they may accumulate in adipose tissue, liver, lungs, spleen, brain, and muscles, with subsequent continued release after the rapid initial reduction of their plasma levels. CBD and THC may reach a steady state with continuous treatments or high doses in a short period (3, 7). *Metabolism*. When used orally, phytocannabinoids show first-pass metabolism. Natural derivatives, such as Δ^9^-THC are metabolized through hydroxylation, decarboxylation, and liver conjugation, by the action of CYP2C isozymes of cytochrome 450. They can also be metabolized in extrahepatic tissues such as the intestine and the lungs. 11-hydroxy-Δ^9^-THC (11-OH-THC), still an active metabolite, is created due to this biotransformation (28–30). There is evidence that THC and CBD can cause interference with the activity of several hepatic cytochrome p450 enzymes' families. Thus, the association of phytocannabinoids with concurrently administered therapies may increase the serum levels of other drugs (28). *Excretion*. Eliminating these compounds by the fecal route includes biliary excretion, so enterohepatic recirculation is possible. Cannabinoids suffer from β- and α-elimination reactions before urinary excretion. Clinical trials in dogs and cats revealed an elimination half-life of 1.0–1.5 h when administered orally, although, in dogs with osteoarthritis (OA) treated with CBD at doses of 2 mg/kg, times of 3.8–6.8 h are reported, which showed no difference when doses >8 mg/kg were used, where the elimination half-life was 3.8–4.8 h (30). Figure 1 summarizes the pharmacokinetics of the phytocannabinoids used in veterinary medicine (10, 18, 29).

The pharmacokinetic parameters can be influenced by the products used as raw materials to prepare the extracts (8, 31). For example, in a study by Deabold et al. (23), the pharmacokinetics of oral (soft chews for dogs and oil for cats) administration of CBD and CBDA obtained from hemp at a single dose of 2 mg/kg (based on CBD) every 12 h for 12 weeks was evaluated in healthy dogs and cats. A mean maximum concentration (Cmax) of 301 ng/mL in dogs and 43 ng/mL in cats, an area under the curve (AUC) of 1,297 ng/h/mL and 164 ng/h/mL, respectively, was observed. The time to reach the maximum concentration of CBD (Tmax) was 1.4 h in dogs and 2 h in cats, indicating a significant difference in the pharmacokinetic parameters between these species (23). These pharmacokinetic differences also became apparent when a transdermal CBD-CBDA-rich extract was used in dogs, where serum concentrations of CBD, CBDA, THC, and its acid derivative tetrahydrocannabinolic acid (THCA) were examined. A 4 mg/kg dose of total cannabinoids twice daily resulted in appx 10 ng/ml of CBD, 21–32 ng/ml of CBDA, trace amounts of THCA, and unquantifiable amounts of THC in serum at the end of 1–2 weeks of treatment, concluding that CBDA and THCA were absorbed better systemically (32). In escalated doses, cannabis oils, including a CBD-predominant oil (2.8–30.5 mg/kg CBD + 0.1–1.1 mg/kg THC), a THC-predominant oil (3.8–41.5 mg/kg THC) and a CBD/THC-predominant oil (1.2–13 mg/kg CBD + 0.8–8.4 mg/kg THC) were studied in cats (33). An interaction of CBD and THC was observed, with higher plasma cannabinoid and metabolite levels following the administration of CBD/THC combination products. Cats appear to have lower serum concentration and faster CBD elimination than dogs (33). Other clinical studies on the pharmacokinetic characteristics of CBD are summarized in Table 1 (23, 30, 31, 34–37).

**Table 1:** Pharmacokinetic studies with different pharmaceutical forms of CBD.

**Pharmaceutical form**	**Absorption kinetics**		**Distribution kinetics**	**Species**	**Reference**
	**Dose**	**Evaluated parameters**			
Oral CBD Hemp based-product (oil)	2 mg/kg total CBD concentration orally twice daily for 12 weeks	Pharmacokinetics parameters Serum chemistry and complete blood counts showed no clinically significant alterations; however one cat showed a persistent rise in alanine aminotransferase (ALT) above the reference range for the duration of the trial. Cats absorb or eliminate CBD differently than dogs, showing lower serum concentrations and adverse effects of excessive licking and head-shaking during oil administration	Cmax) of 301 ng/mL and 43 ng/mL, area under the curve (AUC) of 1,297 ng-h/mL and 164 ng-h/mL, and time to maximal concentration (Tmax) of 1.4 and 2 h, for dogs and cats, respectively	Dogs and cats	Deabold et al. (23)
CBD oil	2 mg/kg oral 8 mg/kg oral	Single-dose pharmacokinetics was performed using two different doses of CBD enriched in osteoarthritic dogs. Each treatment lasted for 4 weeks with a 2-week washout period. The Veterinary assessment showed decreased pain during CBD treatment (*p* < 0.02). Serum chemistry showed an increase in alkaline phosphatase during CBD treatment	Cmax of CBD oil was 102.3 ng/mL (60.7–132.0 ng/mL; 180 nM) and 590.8 ng/mL (389.5–904.5 ng/mL; 1.2 uM) and was reached after 1.5 and 2 h, respectively, for 2 and 8 mg/kg doses Elimination half-life of 4.2 h at both doses and no observable side effects. Clinically, canine brief pain inventory and Hudson activity scores showed a significant decrease in pain and an increase in activity (*p* < 0.01) in patients treated with CBD oil	Dogs	Gamble et al. (30)
Three oral forms of CBD-rich hemp extract Form 1 (Oil A) being a mix of 25% medium-chain triglycerides and 75% long-chain triglyceride. Each milliliter contained 28 mg of CBD, 29 mg of CBDA, 1 mg of THC, 0.8 mg THCA, 0.7 mg of cannabigerolic acid (CBGA), and 1.3 mg of cannabichromene (CBC). Form 2 (Oil B), 25% of the base oil was from sunflower lecithin; 75% of organic sesame oil as Form 1. Form 3, contained ~5 mg of CBDA and 5 mg of CBD in each soft chew.	2 mg/kg of CBD/CBDA (~1 mg/kg CBD and ~1 mg/kg CBDA). Dogs were dosed every 12 h for 2 weeks	CBD, CBDA, THC, and THCA. In addition, metabolized psychoactive component of THC, 11-hydroxy-Δ^9^-tetrahydrocannabinol (11-OH-THC) and CBD metabolites 7-hydroxycannabidiol (7-OH-CBD) and 7-nor-7-carboxycannabidiol (7-COOH-CBD)	No differences were noted for Tmax, T½, AUC and MRT. Significant difference in CBD. Form 3 have a higher Cmax than Form 2, but not Form 1 (*p* = 0.03). Values were 226 vs. 124 ng/ml respectively THC concentrations could not be compared over the 24-h time period due to insufficient data THCA had extensive absorption and higher serum concentrations that were statistically significant between forms. Tmax of 3.3 ng/ml (form 3) vs. 2.2 ng/ml (form 2) and 1.7 ng/ml (form 1). The 7-COOH-CBD concentrations could be compared between Form 2 and Form 3, showing significant differences in Cmax being slightly higher for Form 3 over Form 2 (*p* = 0.02) The metabolites of THC, 11-OH-THC, and COOH-THC-Glu were all below the lower limit of quantitation (1 ng/mL for THC or 2.5 ng/mL for 11-OH-THC and COOH-THC-Glu) A partial lecithin base provides superior absorption and/or retention of CBDA and THCA. No significant changes were observed in ALP, ALT, AST, albumin, total bilirubin, cholesterol and glucose	Dogs	Wakshlag et al. (31)
CBD IV CBD oral	45 mg IV 90 mg IV 180 mg, oral	Doses of 45 to 90 mg generated a proportional increase in AUC, indicating that the pharmacokinetic profile of CBD was not dependent on the administered dose. Three dogs did not record CBD concentrations in plasma after oral administration, and the oral bioavailability was 13–19% in dogs where absorption was observed	CBD IV had a terminal half-life of 9 h, with a triphasic decrease in plasma levels. Plasma clearance was 17 liters/h (at 45 mg doses) and 16 liters/h (after 90 mg). The liver extraction rate was 0.74. Vd of 100 liters	Dogs	Samara et al. (34)
Oral CBD- infused oil Oral microencapsulated CBD-oil beads CBD-infused transdermal cream	75 or 150 mg/ 12 h/ 6 week	75 mg dose: Relative bioavailability of 100, 70.1 and 8.6 % for each formulation, respectively Cmax 625.3; 346.3 and 74.3 ng/mL, respectively 150 mg dose: Bioavailability of 100, 54.7 and 9.9 % for each formulation used respectively Cmax 845.5; 578.1 and 277.6 ng/mL, respectively	75 mg dose MRT of 217, 353 and 490 min average for each formulation used respectively T1/2 of 199.7 y 95.4 min for infused oil and microencapsulated oil. In transdermal cream this was not determined 150 mg dose MRT of 298, 332 and 464 min average for each formulation used, respectively T1/2 of 127.5 y 115.9 min for infused oil and microencapsulated oil. Likewise, in transdermal cream was not determined	Dogs	Bartner et al. (35)
Sativex^®^	Sublingual administration of single doses of 3 consecutive sprays Doses of 3 sprays daily for 14 days	Cmax Δ^9^-THC = 18.5 ng/ml) and Cmax CBD = 10.5 ng/ml, both at 2 h post-administration in the single dose condition. Cmax Δ^9^-THC = 24.5 ng at 1 h post-treatment AUC in a single dose was 94.9 ng/ml/h for Δ^9^-THC and 60.4 ng/ml/h for CBD, observing a similar profile after 14 days of treatment when multiple doses were used. Possible progressive accumulation of CBD and Δ^9^-THC was detected after repeated exposure	Metabolite 11-Hydroxy-Δ^9^-THC produced in the liver from Δ^9^-THC, was almost undetectable, with values for AUC = 6.8 ng/ml/h, Cmax = 1.2 ng/ml and Tmax = 2 h when single dose was used. While AUC = 18.2 ng/ml/h, Cmax = 2.2 ng/ml and Tmax = 2 h in dogs that received multiple doses	Dogs	Fernández- Trapero et al. (36)
Cannabis herbal extract (1:20 THC-CBD)	Single-dose oral at low, medium, or high doses [2, 5, or 10 mg CBD and 0.1, 0.25, or 0.5 mg THC/Kg, respectively	Dogs were monitored for adverse events for up to 48 h post-dose. Evaluations of neurological signs, clinical laboratory abnormalities, and other adverse events were performed in two separate study phases: a multiple-dose phase with 12 dogs receiving five medium doses (5 mg CBD/kg bw) at 12 h intervals and a single low-dose (2 mg CBD/kg bw)	CBD, THC, CBC, and metabolites 6-OH-CBD, 7-OH-CBD, 11-OH-THC, and THC-COOH were quantified in the plasma until 48 h post-administration. CBD and THC: Tmax of 1.9–2.3 h and T1/2 of 2.3–2.6 h. A prolonged elimination phase (CBD T1/2 of 13.3–24.4 h) was observed CBD and THC concentrations increased in a dose-dependent (non-linear) manner. Neurological signs (hyperesthesia or proprioceptive deficits) were noted in 5/6 dogs in the high-dose group but only occasionally or rarely in the medium- and low-dose groups. No clinically meaningful changes in blood count or chemistry values occurred after multiple CHE doses	Dogs	Chicoine et al. (37)

AUC, area under the curve; CBD, cannabidiol; Cmax, maximal concentration; IV,intravenous; MRT, mean residence time; T1/2, half-life; Tmax, mean observed time of maximum concentration; Vd, volume distribution.

## Pharmacodynamics and receptors in the endocannabinoid system

The endocannabinoid system is a signaling pathway found in most vertebrates (30) and regulates various body functions (38). This system consists of three main components: endogenous ligands (anandamide and 2-AG), G-protein coupled receptors and enzymes that degrade and recycle the ligands (39). These enzymes are widely distributed in the organism in levels called the “endocannabinoid tone,” which varies according to the tissue involved (40).

In 1992, Anandamide (N-arachidonoyl-ethanolamine), 2-AG, and 2-arachidonyl glyceryl ether (2-AGE), O-arachidonoyl ethanolamine (virodhamine), and N-arachidonoyl dopamine (NADA) were identified as endogenous agonists of cannabinoid receptors (35). These molecules are locally produced in the cell membrane by hydrolysis of polyunsaturated fatty acids (41). When metabotropic glutamate receptors are activated or in response to an increase in intracellular Ca^2+^, they are released from postsynaptic neurons due to depolarization (3).

**Figure 2:**
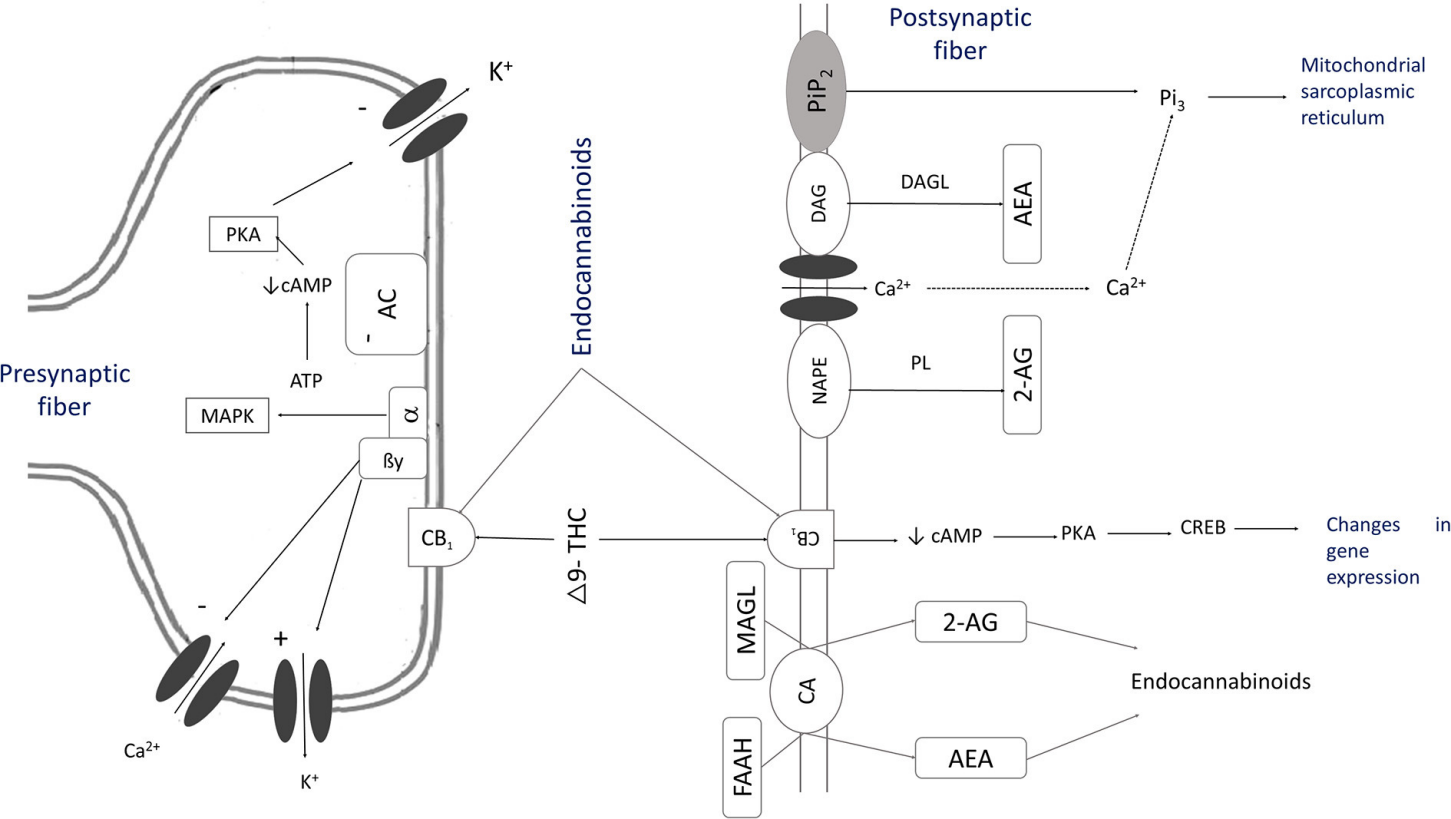
The mechanism of action of cannabinoids [Adapted from (10, 18, 29, 40)]. As a result of the activation of inositol 1,4,5-triphosphate, there is a transient increase of intracellular ionized Ca2+ through the activation of ion channels that synthesize endogenous cannabinoids. This process causes the stimulation of phospholipase (PL) and the hydrolysis of N-arachidonoyl phosphatidylethanolamine (NAPE) to create anandamide (AEA). Phospholipase C (PLC) by phosphatidylinositol 4,5-bisphosphate (PIP2) to diacylglycerol (DAG) and inositol 1,4,5-triphosphate (IP3) and diacylglycerol lipase (DAGL) synthesize 2-arachidonoylglycerol (2-AG). These substances, THC or CBD, activate CB1 receptors. AEA is released into the extracellular space by a membrane transport, and then it is hydrolyzed to become arachidonic acid and ethanolamine by fatty-acid amide hydrolase (FAAH). Specific membrane carriers can also carry 2-AG and hydrolyze it with monoacylglycerol lipase (MAGL) into arachidonic acid and glycerol. This reaction activates Gi/o proteins that stimulate mitogen-activated protein kinases (MAPK), which inhibit adenylate cyclase (AC). The secretion of cyclic adenosine monophosphate (cAMP) is inhibited, hinders voltage-dependent Ca^2+^ channels and stimulates K channels, allowing a G protein (GIRK) flow. The levels of Camp decrease, as does the activation of protein kinase A (PKA), which causes a decrease in the phosphorylation of voltage-gated K channels.

Anandamide and phytocannabinoids are competitively bound to cannabinoid receptors in the presynaptic and postsynaptic membranes of the neurons found in astrocytes, oligodendrocytes, and microglia cells. They can modulate the postsynaptic neuron's excitability in the cell membrane (42, 43) and stimulate Gi/o proteins and mitogen-activated protein kinases (MAPK), causing the inhibition of adenyl cyclase and voltage-dependent Ca^2+^ channels (Figure 2) (10, 18, 29, 40). This mechanism reduces the release of noradrenaline, acetylcholine, glutamate, GABA, glycine, aspartate, serotonin (5HT), dopamine, cholecystokinin (9, 44), and secretion of dynorphins and β-endorphins (16, 45).

Evidence shows that endogenous cannabinoids are linked to a ubiquitous regulating system (30) that involves a group of CB1 (cannabinoid binding 1) and CB2 (cannabinoid binding 2) receptors bound to protein G (GPCR) type Gi/o (39, 46). Once activated, they will retrogradely inhibit neurotransmitters such as gamma-aminobutyric acid (GABA) and glutamate. Other G protein-associated receptors are GPR3, GPR6, GPR12, GPR18, GPR55, and GPR119.

Studies show that the endocannabinoid system is expressed and linked to the nociceptive pathway, where receptors can be found in ascending and descending pain fibers (47). These receptors have also been found in the CNS in presynaptic and postsynaptic GABAergic neurons on the dendritic and somatic surface area of the cerebellum, hippocampus, cerebral cortex, and spinal cord, structures involved in pain modulation (21, 48). G protein-coupled receptor 55 (GPR55 or CB3) is also found in the CNS, but to a lesser extent than CB1 (49).

On the other hand, CB2 is mainly expressed in peripheral tissues and immune and glial cells (3). As a result, they could be involved in sensitizing nociceptive fibers when an immune response has been triggered (50). Studies in rats and dogs have shown the presence of receptors termed peroxisome proliferator-activated receptor alpha, gamma, and beta (PPARα, PPARγ, PPAR_β_), transient receptor potential ankyrin 1 (TRPA1), vanilloid receptor (TRPV1, TRPV2, TRPV3, TRPV4), transient receptor potential (TPR) channels, and the serotonin 5-HT1A receptor (5-HT1AR) (51, 52).

Research in rodents, dogs, cats, and monkeys has shown cannabinoid receptor subtypes, CB1, CB2, and GPR55 (cloned in 1990, 1993, and 1995 respectively), linked to G protein-coupled receptors (18, 46). However, differences have been found among them, such as their number of amino acids, tissue distribution, and sensitivity to specific agonists and antagonists that show selectivity for one or the other receptor (14).

CB1, CB2, and GPR55 receptors identified in dogs have led to *Cannabis sativa* extracts for effective pain control, such as Δ^9^-THC and CBD (16). It has been suggested that the lipophilic properties of cannabinoids enable them to easily cross the blood-brain barrier (53, 54) and cause analgesia, thus rendering them effective in treating pain. Nonetheless, there is still some debate about whether the pain control mechanism of cannabinoids occurs as a result of the CB1 and CB2 receptors agonism or due to the effects caused by the interaction with neuromodulators and the inhibition of neurotransmitters such as glutamate, dopamine, prostaglandins, acetylcholine, GABA, histamine, noradrenaline, and endogenous opioid peptides involved in pain modulation for dogs and cats (3, 55, 56).

It has been reported that cannabinoids can decrease tolerance and maintain response to other drugs after repetitive administration, such as opioids used to treat chronic and acute pain in rodent models (57, 58). As a result, the use of cannabinoids has been suggested as multimodal pain management without adverse effects in the gastrointestinal tract or glomerular filtration (57–59). Doses should be decreased when cannabinoids are combined with other drugs that work through calcium channels (such as gabapentin) to avoid excessive sedation (7). Cannabinoids should be avoided in pregnancy, nursing animals, and animals under 8 weeks of age (60). Controlled clinical trials and research focus mostly on innovative and emerging pain treatments, particularly for chronic and neuropathic pain (28, 61), since cannabinoids can prevent and control central and peripheral sensitization (62). It has been shown that they increase comfort and physical activity in dogs with OA, proving to be an effective treatment for chronic pain (30, 60, 63, 64).

## Spatial distribution of cannabinoid receptors

In the CNS, a high density of cannabinoid receptors has been found in the cerebellum, brain stem, and medulla oblongata (65, 66). However, CB1 can be found primarily in the spinal cord, periaqueductal gray, basal ganglia, cerebellum, and cerebral cortex (21, 67). Subsequent studies in animals using immunohistochemistry have demonstrated the presence of CB1 receptors on the axon terminal in the presynaptic and postsynaptic membrane in astrocytes, oligodendrocytes, microglial cells, meninges (the dura mater), and the cerebral cortex (frontal lobe, neocortex, and gray matter), where this receptor is related to memory and association processes. Other sites where these receptors have been found to a lesser extent are the amygdala, nucleus accumbens, hypothalamus, thalamus, cerebellum, and midbrain (periaqueductal gray); these receptors participate in central antinociceptive and analgesic effects (12, 68). Receptors can also be found in the medulla oblongata, with a higher density in the spinal trigeminal nucleus and the olfactory bulb, where there is a higher expression, and their activation could be associated with the modulation of food intake. Receptors have also been identified in the parabrachial nucleus, areas of the brain stem, and the spinal cord (dorsal horn of lamina X and the ventral horn in the cervical, thoracic, and lumbar segments), some Purkinje cells, peripheral nerves, the outermost part of the skin, fibroblasts, and macrophages (39, 52, 69–71).

The retrograde inhibition of neurosecretion of acetylcholine, dopamine, GABA, histamine, 5HT, glutamine, cholecystokinin, D-aspartate, glycine, and noradrenaline has established that CB1 receptors are predominantly found in presynaptic fibers (56, 71, 72). Consequently, agonists of this type of receptor display more psychoactive adverse reactions in the CNS (hypothermia, ataxia. or euphoria), thus having a minor role in pain control despite various animal models studies show that they inhibit the production of cyclooxygenases (73). On the other hand, their use to treat seizures in dog and rodent models has increased. Cerebrospinal fluid samples were obtained from dogs suffering from idiopathic epilepsy, where an increased level of anandamide and endocannabinoid was observed compared to healthy dogs (74).

The expression of CB2 receptors is found in high densities in immune cells such as T and B lymphocytes, CD4, and CD8, which inhibits the release of interleukin th2, 10, 12, and interferon-gamma, natural killer cells, mast cells, macrophages, and neutrophils (14, 75). These receptors have been identified in the spleen, pancreas, thymus, lungs, tonsils, parotid and mandibular glands, Peyer's patches, basal ganglia, enteric neurons, striatum, synovial membrane, skin keratinocytes, as well and endothelial cells in blood vessels (4). As part of the CNS, they can be seen in the amygdala, hippocampus, cerebellum, cerebral cortex, nucleus accumbens, globus pallidus, and striatum (76, 77). In addition, these receptors are expressed in microglia and astroglia. Thus, this discovery has given rise to treatments for neuroinflammatory diseases based on therapeutic targets (51). There has been a debate surrounding the psychotropic ability of some cannabis extracts or synthetic derivatives due to the activation of CB1 receptors. However, it is unclear if CB2 receptors play a role in this process (3, 5, 26).

The expression of these receptors in dogs and cats made it possible to identify several receptors, such as GPR55, in the gastrointestinal tract, specifically the mucus (lamina propria and epithelial cells) and muscular layers, the stomach, pylorus, and colon. The PPARα, PPARγ, and TRPV1 receptors have been found in the lamina propria of the pylorus, duodenum, ileum, colon, enterochromaffin cells in the stomach, and endothelial cells in blood vessels. PPAR receptors have been associated with antinociceptive and anti-inflammatory effects and the prevention of hyperalgesia and allodynia. Alpha receptors have been used as therapeutic targets for treating allergy-related or eosinophilic skin diseases in dogs and cats (51, 52, 78, 79).

Therefore, the cannabinoid system regulates physiological and pathological processes in humans and animals and several tissues' nociceptive reflexes and inflammatory processes. As a result, the use of natural or synthetic cannabinoids inhibits the secretion of neurotransmitters and ions responsible for the modulation, projection, and perception of pain (72, 80), and this use in companion animals has become an object of study (67–70).

## The role of cannabinoids in pain modulation

Acute, chronic, and neuropathic pain management uses new substances that assist traditional analgesics, such as NSAIDs, opioids, local anesthetics, and a ketamine. Therefore, cannabinoids are suggested as a complementary and effective alternative (7). To manage pain, drugs that intervene in the nociceptive pathway should be used as part of a multimodal protocol (20, 81), which is seen as a therapeutic advantage for the endocannabinoid system, as it is expressed in the ascending (perception) and descending (modulation) pathways of the periphery and terminal central of primary afferents (47). It can modulate stimuli in Rexed laminae I, II, and X of the dorsal horn in the spinal cord, where high concentrations of CB1 receptors can be found (71).

Several authors have reported that histological analysis, immunohistochemistry, and immunofluorescence revealed immunoreactivity in the cerebral cortex, cornu ammonis, and dentate gyrus of the hippocampus, where cells that react to CB1 were found, proving the presence and participation of cannabinoids in chronic pain control and patients with epilepsy (7, 58). These findings confirm that synthetic agonists found in cannabinoids or endocannabinoids prevent central sensitization due to the inhibition of GABA, glutamate, and voltage-dependent channels (72, 73, 82).

The selective activation of CB2 receptors has shown antinociception, anti-inflammatory, and neuroprotective effects in animal models such as rodents, dogs, and monkeys with OA, inflammation, spinal cord injury, and neuropathies (7, 28). As they activate antiallodynic effects, they inhibit the hyperactivity of primary afferent fibers and decrease the release of neurotransmitters that act on nociceptors (28, 72, 83). This expression on the dorsal horn of the spinal cord creates a positive regulation in the presence of neuropathic and inflammatory pain (84, 85). As a result, this would confirm that in inflammatory lesions that affect nervous tissue, there is modulation by CB2 receptors.

The CB2 receptor is involved in immune processes by inhibiting the release of cytokines, thus, controlling oncological pain and preventing the progression of neuropathies, OA, arteriosclerosis, and neurodegenerative and neuroinflammatory diseases (86). Also, degenerative myelopathies, meningitis-arteritis, spinal spirocercosis, and epilepsy. An example can be found in patients with spinal cord injuries with higher activity and the presence of CB2 receptors on microglia (87).

The mechanisms that enable the CB2 receptors to produce analgesia involve several signaling pathways with cyclic AMP (AMPc), nerve growth factor (NGF), mitogen-activated kinase (MAP kinase), nuclear factor kappa B (NK-KB), calcium *via* JAK-STAT 1, ceramides, caspases, and c-Jun N-terminal kinases, which can be activated by second messengers such as Gi/o proteins (14, 36). A mechanism is still under research consisting of using synthetic agonists in mice, such as AM1241, which stimulated the release of β-endorphins that can activate μ-opioid receptors (46).

The involvement of CB1 and CB2 agonists in inflammation and pain reveals a promising therapeutic target. However, despite the evidence of its use and effectiveness in animals and humans, its effects are still under research. The role of CB1 and CB2 receptors is still an object of study since it can be associated with several intracellular mechanisms that modulate nociception (51, 88).

CBD has at least 76 different molecular targets of action. For example, it exerts its pain-relieving effects by interactions and modulation of inflammatory and nociceptive systems (as TRPV1 reverse agonist or COX2 inhibition). CBD has high activity in other ionotropic transient receptors potential channels, such as TRPA1, TRPV4, TRPV2, and TRPM8. Other groups of receptors that CBD binds are Gi-coupled receptors (GPR55, GPR18), PPARs, as opioid, 5-HT, or dopamine receptors (89, 90).

## Efficacy of CBD in controlling pain

CBD has been studied to control pain in companion animals in chronic pain models, mainly related to OA (12, 30, 46, 63). Table 2 summarizes some clinical studies regarding this use (30, 63, 64, 91–93). Valastro et al. (62) identified and quantified endocannabinoids in synovial fluids of dogs with OA. They concluded that osteoarthritic knees had higher 2-AG concentrations, as oleoylethanolamide (OEA) presence, when compared with contralateral joints. This study tested the effect of modulating pain and inflammation by describing the presence of endocannabinoid receptors in synovial fluids and adjacent inflamed tissues. This aspect is why cannabinoids effectively treat osteoarthritic pain in dogs, as mentioned by Brioschi et al. (63) and Kogan et al. (91), although in the first case, it was in multimodal therapy. However, the efficacy of CBD is still under discussion, as Mejia et al. (94) mentioned when measuring the safety and effect of CBD in controlling signs associated with pain caused by canine OA for 6 weeks. The authors concluded that there was no significant difference in the assessment of animals' gait and activity level of animals, describing that CBD as monotherapy does not have an appropriate analgesic efficacy to control pain. In contrast, some authors suggest that CBD or THC analgesic properties are dose-dependent (95, 96).

**Table 2:** Clinical studies of the use of CBD in chronic pain models in dogs and cats.

**Pain model**	**Compound**	**Species**	**Route**	**Dosage**	**Period**	**Results**	**Reference**
OA	CBD oil	Dog	Oral	2 mg/kg/12 h	4 w	Based on the Hudson Activity Scores, the activity level increased. Pain was significantly decreased, as was shown by the Canine Brief Pain Inventory scoring system (*p* < 0.01). Concomitant use of NSAIDs reduced lameness scores (*p* = 0.03). Veterinary pain scores showed a decrease from baseline in dogs on NSAIDs (*p* < 0.01)	Gamble et al. (30)
OA	CBD oil	Dog	Oromucosal	2 mg/kg/12 h	12 w	According to the Canine Brief Pain Inventory scoring system, the addition of CBD to anti-inflammatory drugs (firocoxib or prednisone), gabapentin and amitriptyline (multimodal therapy) reduced the pain severity (*p* = 0.0002 to 0.016) and increased the Quality-of-Life index (*p* = 0.003)	Brioschi et al. (63)
Lameness (OA)	Naked CBD Liposomal CBD	Dog	Oral	0.5 mg/kg or 1.2 mg/kg for naked CBD or 20 mg/day liposomal CBD (three groups)	4 w	Significantly decreased pain (*p* ≤ 0.01) and increased mobility (walking, running, and standing position) in a dose-dependent association (50 mg/kg naked CBD or 20 mg/kg liposomal CBD), and the effect remained 15 days after cessation of therapy	Verrico et al. (64)
Chronic maladaptive (OA)	CBD oil	Dog	Oral	0.25 mg/kg/12 h 0.5–0.75 mg/kg/ 12 h	90 d	Using the Cincinnati Orthopedic Disability index as a pain assessment every 2 weeks, CBD reduced the pain scores from 3.2 ± 2.2 to 0.97 ± 0.81 Additionally, gabapentin doses were reduced by 20 to 40% in some dogs	Kogan et al. (91)
OA	CBD	Dog	Oral	1 mg/kg/12 h	30 d	From day 3, the pain was reduced by 32% (81 to 54 points by day 30), according to the Canine Brief Pain Inventory in dogs	Furtado de Álava (92)
Sarcoma	THC/CBD oil	Cat	Topical oral	0.2 mg/kg/12 h 0.05 cc	10 d	Extracted cannabis oil diluted with normal saline (1:5) reduced the pain caused by the sarcoma growth and reduced its size from 5 to 1.5 cm	Buranakarn (93)

d, days; h, hours; w, weeks; OA, osteoarthritis.

## Adverse effects of cannabinoids

Cannabis intoxication is considered a clinical condition with a good prognosis in dogs as long as there are no comorbidities, complications, or co-ingestion of other potentially intoxicating substances. It is mentioned that the LD50 (median lethal dose) of oral THC in dogs appears to be >3 g/kg. Regarding CBD, there are no published data on its LD50, so it can be inferred that until now, in controlled clinical studies, this phenomenon has not been observed (12). However, regarding clinical safety and adverse effects in multidose administration schemes, it has been reported that high doses of 10 mg CBD + 0.5 mg THC/kg and medium doses of 5 mg CBD + 0.25 mg THC/kg; CBD metabolites were detected up to 48 h later. It also shows slow elimination phases accompanied by signs such as hyperesthesia (auditory/visual/tactile stimuli) and proprioceptive deficits in the first 2 h of administration, which disappeared within 4–6 h after the presentation (37). Other adverse events observed in the high-dose group included ptyalism (1/6 dogs), urinary incontinence (1/6 dogs), and vomiting (3/6 dogs). On the other hand, animals treated with single and multi-dose neurological changes (mydriasis, ataxia, hyperacusis, and delayed reflex responses) were detected 2 h after administering the single dose of 5 mg CBD/kg. However, the signs significantly decreased or were absent within the first 6 h (37).

On the other hand, Brioschi et al. (63) report the presence of minimal ptyalism in 22% of the animals treated with CBD (2 of 9 dogs), in addition to somnolence and mild ataxia in 1 of 9 experimental subjects. Similarly, Vaughn et al. (97) found mild adverse effects in 94.9% of the cases, moderate effects in 4.4% of the animals under study, and only 0.8% had severe effects, such as lethargy, hypothermia, and ataxia. It is worth mentioning that these effects were attributed to a THC preparation, which implies that these products' chemical and pharmacokinetic characteristics could be the main responsible for the appearance of adverse neurological signs. Likewise, in a similar study in dogs medicated with a repeated dose of 12 mg/kg of a cannabis extract orally for 28 days, mild gastrointestinal signs such as hypersalivation were generated, in addition to an increase in serum alkaline phosphatase (98). This reveals that these adverse effects are related to the exposure time, type of product, and dose used.

Janeczek et al. (99) reported the adverse effects of phytocannabinoid intoxication in a cat, describing consciousness disorders, seizures, ataxia, depression, anxiety, vocalization, hypersalivation, diarrhea, vomiting, bradycardia or tachycardia, hypothermia, and mydriasis (99). From a comparative point of view, these effects are similar to those reported in dogs. However, according to Kulpa et al. (33), the safety and tolerability of oral staggered doses of cannabis oil produce mild and transient adverse effects, even though the administration of CBD oil was carried out at a dose of 30.5 mg/kg were lethargy, hypothermia, ataxia, and nictitating membrane protrusion were observed, which did not require medical intervention for resolution. Unfortunately, the lack of related information on this species is limited, making it difficult to compare the incidence of adverse effects reported in dogs and corroborate its safety in cats. In such a way, it is necessary to emphasize that despite the clinical usefulness of cannabinoids, the present adverse effects cannot be ignored, which must be controlled and resolved by the veterinarian during their use.

## Study perspectives, limitations of its clinical application, and therapeutic evaluations of the use of cannabinoids in rodents models, dogs, and cats

Studies focusing on cannabinoids used in veterinary medicine predominate in laboratory animals rather than domestic species, particularly dogs and cats. These studies have enabled the development of synthetic agonists of cannabinoid receptors based on their chemical structure and further research regarding the specific distribution of cannabinoid receptors in the CNS. Due to the limitations of these drugs, they are currently being used only *in vitro* and in test animals such as mice, rats, or guinea pigs to transfer them to human medicine (88). However, WIN55,212–2, a synthetic agonist, has been used as an anticonvulsant, and it is aimed at dogs suffering from idiopathic epilepsy that have shown increased levels of anandamide and endocannabinoids in their cerebrospinal fluid, compared to healthy animals (67).

Gamble et al. (30) recently tested CBD oil's safety and analgesic efficiency at a concentration of 10 mg/mL of CBD **(**as an equal mix of CBD and CBDA) and 0.24 mg/mL; 0.27 mg/mL of THC and cannabichromene respectively, in dogs with chronic pain caused by OA. Each dog received a 2 mg/kg and an 8 mg/kg oral dosage for 4 weeks and showed an elimination half-life of 4.2 h without side effects. The evaluations to assess the analgesic efficiency and Hudson activity scores showed a significant decrease in pain degree with the dosage used. During the trial, dogs were only allowed to receive NSAIDs, fish oil, and/or glucosamine/chondroitin sulfate without changing in these medications for 4 weeks before or during the 10-week study period as a standard of care for the disease process. Regarding clinical safety and toxicity tests, no significant difference was noted in BUN, creatinine, or phosphorus between dogs treated with CBD oil vs. the placebo oil, while NSAID treatment resulted in a higher creatinine concentration. In this study, an increase in ALP activity was also reported in nine dogs treated with CBD, explaining that this effect may be due to the induction of oxidative metabolism in the liver mediated by cytochrome p450.

The clinical efficiency of cannabinoids to treat oncological pain in veterinary patients has also been proved after it was shown that nociceptive reactions result from the stimulation of visceral and somatic afferent pathways that could cause neuropathic pain (3, 7). Inflammatory reactions are common in cancer and can be treated with opioids and NSAIDs (100). This condition favors the presence of fatigue, decreased mobility, cognitive disorders, urinary conditions, and paraneoplastic syndrome-related pain (moderate to severe). As a result, using natural derivatives such as cannabinoids offers a safe alternative for pain control. In addition, this neoplastic activity would be beneficial to control adverse reactions caused by antineoplastics or cancer itself (101).

Johnson et al. (102) conducted a follow-up study on 43 human patients with advanced cancer-related pain. A THC/CBD spray was used to assess efficacy and tolerability to opioids for 3 weeks. There was a significant reduction in pain, as seen in the scores from questionnaires that represented pain. No safety issues associated with the use of this spray were reported. In veterinary medicine, CBD has shown promising results as a single prescription or in combination with mitoxantrone and vinblastine for the treatment of canine urothelial carcinoma cells (103), in canine cutaneous mast cell tumor (104) and even as multimodal compassionate pain therapy reported in a case of a dog with OA and testicular neoplasia that was medicated with robencoxib, gabapentin, and a formulation of liposomal CBD injected subcutaneously (105).

Pharmacological interactions like the ones mentioned above have already been studied, and the use of cannabinoids created a synergic effect with opioid agonists (106). Similar studies revealed an analgesic efficacy in rats that were given a combination of subcutaneous morphine along with intraperitoneal administration of THC at high (100 and 4 mg/kg, respectively) and low dosages (75 mg/kg and 4 mg/kg), both of which produced analgesia. In addition, this combination prevented tolerability to opioids; these results are similar to other studies that have used CB receptor agonists (57–59). An explanation for this synergic effect is that both drugs produce analgesia through signaling pathways linked to second messengers such as protein G (14, 107).

Regarding the development of pharmaceuticals, the University of Cambridge manufactured a product called Sativex^®^ equivalent to 8.1 mg of Δ^9^-THC:7.5 mg of CBD), which has been used in human medicine and is currently used for controlled clinical tests in animals such as dogs and cats (3, 87).

Additionally, it has been reported that CBD can reduce the consumption of inhalational anesthetics in animal models. In this regard, the administration of CBD and pure opioids such as morphine significantly reduces the consumption of inhalational anesthetics (108). Other studies indicate that a single dose of cannabinoids can modify the anesthetic depth with inhalational agents, where electroencephalographic alterations can be monitored through the bispectral index (BIS) (109). However, a clear discussion is necessary if this benefit could exceed the pain control provided by this substance (110). Regarding other interactions, a potentiation effect with benzodiazepines has been described due to the GABA's binding affinity and degradation. With gabapentin, CBD enhances its analgesic effect, and the doses can be reduced. On the other hand, the action of cannabinoids on 5HT-1A, 5HT-2A, and 5HT-3A receptors can increase the effects of serotonin syndrome (7). Concerning other anesthetics, studies in rats have shown that ketamine can function as an exogenous agonist of CBD, releasing anandamide (110), and propofol inhibits CB1 and CB2-mediated sympathetic responses, so its effect can be prolonged (111), as with barbiturates and alfaxalone (7).

## Conclusions

The study of the endocannabinoid system, its ligands, receptors, mechanism of action, and signaling, has led to research showing that cannabis extracts and synthetic derivatives are an effective therapeutic alternative for the multimodal management of pain. The evidence suggests that cannabinoids can be used in veterinary medicine to treat acute, chronic and neuropathic pain due to their ability to prevent peripheral and central sensitization (112).

However, since the endocannabinoid system is a signaling pathway that regulates several actions, its interaction with different endogenous ligands and neurotransmitter modulation can have beneficial effects on patients suffering from seizures, contact and atopic dermatitis, epilepsy, degenerative myelopathies, asthma, diabetes, glaucoma, retinitis pigmentosa, and inflammatory diseases. These compounds also possess antineoplastic, appetite-stimulating, and antiemetic properties (3, 90, 113–116).

According to some clinical trials, CBD is a potential analgesic drug for chronic pain control in companion animals, although it seems to have a dose-dependent fashion. These results show the importance of studying cannabinoids and their effect on the CNS and PNS and the expression and role of its receptors in companion animals as a potential field of study for veterinary medicine to offer health benefits and wellbeing to future patients.

## Author contributions

All authors listed have made a substantial, direct, and intellectual contribution to the work and approved it for publication.
